# Learning the ropes: strategies program directors use to facilitate organizational socialization of newcomer residents, a qualitative study

**DOI:** 10.1186/s12909-022-03315-9

**Published:** 2022-04-05

**Authors:** Gerbrich Galema, Robbert Duvivier, Jan Pols, Debbie Jaarsma, Götz Wietasch

**Affiliations:** 1 University of Groningen, University Medical Center Groningen, Department of Anesthesiology, Groningen, the Netherlands; 2 University of Groningen, University Medical Center Groningen, Center for Education Development and Research in health professions (CEDAR), Lifelong Learning, Education and Assessment Research Network (LEARN), Groningen, the Netherlands; 3 Parnassia Psychiatric Institute, The Hague, the Netherlands; 4 Faculty of Veterinary Medicine, Utrecht University, Utrecht, the Netherlands

**Keywords:** Faculty development, Newcomer adjustment, Organizational socialization, Postgraduate medical education, Program directors, Transitions

## Abstract

**Background:**

Many residents experience their transitions, such as from medical student to resident, as demanding and stressful. The challenges they face are twofold: coping with changes in tasks or responsibilities and performing (new) social roles. This process of ‘learning the ropes’ is known as Organizational Socialization (OS). Although there is substantial literature on transitions from the perspective of residents, the voices of program directors (PDs) who facilitate and guide residents through the organizational socialization process have not yet been explored. PDs’ perspectives are important, since PDs are formally responsible for Postgraduate Medical Education (PGME) and contribute, directly or indirectly, to residents’ socialization process. Using the lens of OS, we explored what strategies PDs use to facilitate organizational socialization of newcomer residents.

**Methods:**

We conducted semi-structured interviews with 17 PDs of different specialties. We used a theory-informing inductive data analysis study design, comprising an inductive thematic analysis, a deductive interpretation of the results through the lens of OS and, subsequently, an inductive analysis to identify overarching insights.

**Results:**

We identified six strategies PDs used to facilitate organizational socialization of newcomer residents and uncovered two overarching insights. First, PDs varied in the extent to which they planned their guidance. Some PDs planned socialization as an explicit learning objective and assigned residents’ tasks and responsibilities accordingly, making it an intended program outcome. However, socialization was also facilitated by social interactions in the workplace, making it an unintended program outcome. Second, PDs varied in the extent to which they adapted their strategies to the newcomer residents. Some PDs used individualized strategies tailored to individual residents’ needs and skills, particularly in cases of poor performance, by broaching and discussing the issue or adjusting tasks and responsibilities. However, PDs also used workplace strategies requiring residents to adjust to the workplace without much intervention, which was often viewed as an implicit expectation.

**Conclusions:**

PDs’ used both intentional and unintentional strategies to facilitate socialization in residents, which may imply that socialization can occur irrespective of the PD’s strategy. PDs’ strategies varied from an individual-centered to a workplace-centered approach to socialization. Further research is needed to gain a deeper understanding of residents’ perceptions of PD’s efforts to facilitate their socialization process during transitions.

**Supplementary Information:**

The online version contains supplementary material available at 10.1186/s12909-022-03315-9.

## Background

Residents experience many transitions throughout their educational career, not only the transition from student to resident but also transitions between rotations during residency training [1, 2]. These transitions can be demanding and stressful for them for several reasons [3, 4]. First, residents have to adapt to a demanding role within a new context, which involves profound changes in tasks, responsibilities and expectations [5, 6]. Second, residents need to learn how to perform their social roles, find out how to function well in a team and adapt to existing norms and customs. In organizational sciences, these challenges are referred to as Organizational Socialization (OS) which can be defined as ‘a process by which an individual acquires the social knowledge and skills necessary to assume an organizational role’ [7]. In doing so, newcomer residents become part of existing social, cultural and political practices and traditions within a department [8]. Therefore, ‘learning the ropes’ seems to be an important challenge in transitioning from one role to another [9].

Despite a growing interest in transitions in medical education, guidance (by others) to facilitate socialization of residents in transition has rarely been a subject of research. In Health Professions Education (HPE) literature, the topic of socialization has widely been discussed from different perspectives. A commonly used perspective that aims to understand the development of individual newcomers is Professional Identity Formation (PIF) [10, 11]. In this perspective, socialization is seen as one of the driving forces behind the transformation of the individual from a layperson to a skilled professional [11]. Cruess et al. [11] recognized that socialization and thus social interaction between individuals occurs in communities of practice. However, most research [12, 13] solely focuses on the perspective of the individual apprentice. It seems that PIF literature offers limited explanation about how ‘others’ in the community of practice perceive and support the individual apprentice’s socialization process.

The concept of OS offers a theoretical lens to understand socialization from the perspective of both the individual and the ‘other’. As Chao [14] stated, ‘OS is a learning and adjustment process that enables an individual to assume an organizational role that fits both organizational and individual needs’. Based on a meta-analysis of 12.000 graduates in the corporate field, Bauer and Erdogan proposed a general, linear three-phase model of organizational socialization [15, 16]. This model is often visualized as a linear three-phase model describing the socialization processes of a newcomer: phase one refers to factors related to new employee characteristics, new employee behaviors and organizational efforts to facilitate the transition; phase two refers to newcomer adjustment, indicating how well a newcomer in transition is doing; and phase three refers to new employee socialization outcomes [16]. In the field of Health Professions Education, this model has been used in studies on the transition of undergraduate medical students into clinical clerkship [17], and graduate nurse transition [18]. In these studies, the authors suggested to optimize transitions by organizational efforts, such as a formal or informal orientation, a limited number of workplace changes in the first year of practice, mentorships or other documented strategies of social support [17, 18]. Using individual perspectives of undergraduate medical students in their clinical rotations, Atherley et al. highlighted the role of insiders, i.e. faculty, to ensure adequate socialization to smooth the transition into a new clerkship [17]. However, in the context of the transition from student to resident, it has not yet been studied to what extent insiders, such as residency program directors (PDs), facilitate newcomer residents’ transition into clinical practice and how newcomer residents are integrated into clinical teams. The latter refers to phase 1 of the aforementioned organizational socialization model: organizational efforts to facilitate the transition [16]. To shed more light on residents’ socialization processes and advance the understanding on this topic, we investigated program directors’ (PDs) perspectives on the way they facilitate and guide newcomer residents.

In teaching hospitals, PDs play an important role in the socialization process of residents through both frequent interactions during daily work activities and their formal leadership position. In daily practice, PDs are part of the community of practice in which newcomer residents enter. Here, socialization occurs through social interaction between individuals (i.e. members of the health care team), which advances learning [11]. PDs’ formal leadership position comprises responsibility and accountability for the structure, organization and administration of the entire residency program [19]. Despite the role PDs have in the socialization process of newcomer residents and their responsibility and engagement in the residency program, it is yet unknown *how* they support the socialization process of newcomer residents. Insight into PDs’ strategies to foster newcomer residents’ transition and socialization can help reduce stress among residents during transitions [20].

In a first step to explore this gap in the literature, we decided to do a qualitative study to gather rich and meaningful information and explore the full range of what a PD can do to foster socialization. We formulated the following research question: What strategies do PDs use to facilitate organizational socialization of newcomer residents? To answer this research question, we conducted a multi-site, qualitative study using semi-structured interviews and inductive thematic data analysis [21]. Subsequently, we used Organizational Socialization as an analytical lens to describe and better understand PDs’ efforts to facilitate residents’ socialization, because OS encompasses organizational strategies to structure experiences of an individual in transition from one role to another [7]. Finally, we used inductive analysis to identify overarching insights.

## Methods

### Study setting – context

This study was conducted in one academic center and seven teaching hospitals in the Netherlands. We included Program Directors, associate Program Directors and supervisors (from here on named PD) who were members of a dedicated team responsible for Postgraduate Medical Education (PGME) of residents. A PD can be responsible for up to 75 residents. PGME consists of competency-based education that meets national requirements [22] and is structured into specialty-specific national curricula with local training plans. Based on these local training plans, the PD and the resident together create a personal training plan that keeps track of individual competencies and learning needs [22]. PDs need to develop pedagogical competencies that can be obtained through (mandatory) pedagogical training (e.g. train the trainer courses). PDs are supported by managerial / administrative assistants, who look after the placement side of residents’ employment. Every hospital has a central PGME committee consisting of several PDs and residents from different specialties, which is chaired by a dean. The committee is responsible for the quality and collaboration of the different PGME programs within the hospital [22]. In all participating hospitals, PGME committees are supported by educational (policy) advisors and scientists.

### Participants

To ensure participants were able to describe their experiences with newcomer residents in transition, participants were required (1) to work as a PD, associate PD or supervisors in a dedicated team that was responsible for PGME of residents in a hospital-based specialty and (2) to collaborate with newcomer residents on a regular basis. We purposively sampled [23] PDs from different specialties –covering the entire spectrum of surgical, medical and supportive specialties– and different hospitals, to ensure diversity in PGME programs and work environments. This broad sample was intended to elicit rich and meaningful information and a broad variety of descriptions to help answer the research question [24], which could in turn facilitate transferability to other settings [25]. Participants were invited by e-mail, stating the purpose of the study and assuring that all data would be treated confidentially, anonymity would be guaranteed and participants could withdraw at any time [25]. Of the 39 PDs we invited, 17 agreed to participate and were interviewed. Six participants worked at an academic center and 11 worked at different non-academic teaching hospital. Four PDs did not meet the inclusion criteria since they did not collaborate with first-year residents on a regular basis. They suggested to interview other faculty instead, i.e. associate PDs and supervisors who were dedicated to guiding and supporting first-year residents, and were working in a dedicated team with the PD. Characteristics of the participants are shown in Table 1.

**Table 1:** Characteristics of the participants (*n* = 17) interviewed in this study

Attribute	Number of participants
Gender	
Female	2
Male	15
Hospital	
Academic hospital	6
Non-academic teaching hospital	11
Specialty: surgical	
Surgery	4
Obstetrics and Gynecology	3
Specialty: non-surgical	
Internal medicine	5
Radiology and nuclear medicine	2
Pediatrics	1
Anesthesiology	1
Pathology	1
Role	
Program Director	13
Associate Program Director	2
Dedicated supervisor responsible for PGME of first-year residents	2

### Study design, data collection and analysis

#### Study design

We used a theory-informing inductive data analysis study design [26]. We conducted semi-structured interviews to collect data that was relevant to our research question. Data analysis involved three distinct phases: (1) an explorative, a-theoretical, inductive phase, in which we coded the interview transcripts without using a predefined coding scheme and grouped the codes into themes (thematic analysis), which is an appropriate method to ‘identify, analyze, report patterns within the data and helps to identify or examine underlying ideas, assumptions, and conceptualizations’ [21]. (2) a theory-driven, deductive phase, in which we used the analytical lens of Organizational Socialization (OS) tactics [7] to deepen our understanding of the constructed themes and further refine and make sense of the phenomenon studied [27]. (3) in the final phase, we moved from deduction to induction and sought to identify and explicate overarching insights [28].

#### Data collection

In the period April to June 2018, the first author conducted semi-structured interviews with all participants. Two other researchers (JP, MB) accompanied and observed the first author in 4 interviews and the remaining interviews were conducted by the first author alone. Including these two researchers in the interview process contributed to crystallization, as their perspectives provided us with a more complex, in-depth, but still thoroughly partial understanding of the issue [24]. The interviews were guided by an interview guide (Additional file 1). To ensure rigor [24] GG made field notes after every interview and debriefed with JP and GW to discuss the responses of the interviewees. The debriefings provided additional insights into un- or underexplored topics that seemed relevant to the research question. The interview guide was rephrased accordingly and un(der)explored areas were elaborated on in consecutive interviews [23] as an iterative part of the research cycle [25]. The interviews lasted 35 to 90 min, were recorded and transcribed verbatim using F4transkript [29]. Data collection was continued until no new information was obtained from PDs’ explanations of how they facilitated newcomer residents’ transitions.

#### Data analysis

Data analysis consisted of 3 phases, as described above (inductive – deductive – inductive). Phase 1 (inductive): We performed thematic analysis using the steps proposed by Braun et al. [21]. First, two authors (GG and JP) familiarized themselves with the data. Then, the first author inductively coded all interviews line by line, without trying to fit it into a pre-existing coding frame [21]. Next, GG and JP moved from coding to searching for initial themes. Semantic analysis showed the PDs’ perspectives of tasks residents performed during the first period of their new job. After that, the initial themes were reviewed within the team and we noticed that the PDs also focused on the experiences of residents in transition. We became interested in the examples the PDs mentioned to illustrate their support to residents in this phase of training. Therefore, we decided to expand our scope from solely focusing on residents’ tasks to including PDs’ underlying ideas, assumptions and conceptualizations [21, 27] about newcomer residents’ transition, and their support during the transition period. Phase 2 (deductive): To further shape and define the initial themes, two researchers (GG and RD) deductively compared the data [30] to the descriptions of van Maanen & Schein’s [7] concepts of OS tactics, which will be described in more detail in a separate section below. Phase 3 (inductive): To get richer insights, we used an inductive approach and synthesized our findings to uncover overarching insights [28].

Throughout the analytical process, the entire research team held regular meetings to review the coding process, discuss the data interpretation and reach a mutual understanding of codes and themes. The team meetings, therefore, contributed to the analytical process and ensured the credibility and the consistency of the interpretation. The first author maintained an audit trail to keep track of the team’s thinking process and document analytic decisions. Qualitative data analysis was supported by Atlas.ti, version 8 [31].

### Analytic framework

We used OS theory as a lens to further interpret the findings of our thematic analyses, in particular the description of socialization tactics. These tactics are characterized by how others in the organization let newcomers adjust to their new role. This teaching and learning process is referred to as the OS process. Each tactic is represented as a distinguishable set of events that affect individuals in transition. The overarching goals of the OS process is the sustainability of the organization, through transmission of values and information [7]. Van Maanen & Schein proposed 6 different OS tactics, which are often used in empirical research in organizational literature [32–34]. A summary of the tactics is provided in Table 2. Each tactic is illustrated by describing the underlying two opposites.

**Table 2:** Description of Organizational Socialization Tactics; summary of Van Maanen and Schein [7]

Tactic	Description
Collective and individual socialization tactic	The degree to which newcomers are socialized in a group with common experiences, or separated from other newcomers so they have ‘a more or less unique set of experiences’
Formal and informal socialization tactics	Whether newcomers participate in a structured program tailored to their role of newcomer, separated from regular employees, or in a program that does not distinguish the newcomers’ role from other roles, so they learn their new role through trial and error
Sequential and random socialization tactic	The degree to which the organization plans the socialization as a gradual process or more random, when the sequence of steps is unknown or ambiguous
Fixed and variable socialization tactic	The degree to which the organization expects that socialization occurs within a fixed timeframe, or more variable giving newcomers few cues as to when to expect a given boundary passage
Serial and disjunctive tactic	The degree to which newcomers are socialized with the help of role-models, or not
Investiture and divestiture socialization tactic	The degree to which organizations build upon the capabilities and values newcomers acquired previously and affirm their gained self-image, or deny and strips away certain newcomer characteristics and rebuild newcomers’ self-image

### Reflexivity

From our constructivist perspective, we are aware that realities are socially constructed [35] and that our interests, perspectives and backgrounds shaped the research and interpretation of the data throughout the study [24, 36]. All members of the research team have been experiencing transitions: from school to work and from one job to another, which shaped our thoughts in this research process. Two researchers were experienced in newcomer transitions from the leadership perspective. In their respective roles as PD (GW) and leader of a Research Network (DJ) they supported many newcomers in their first job. Three researchers, a resident in anesthesiology (GG), a resident in psychiatry (RD) and a PD of anesthesiology (GW), worked in the researched context. Their experiences helped the entire team understand PDs’ views and terminology, and embed PDs’ descriptions into the lived experience [28, 37]. However, a shortcoming of being an insider and too close with the participants in research could be that we as researchers assume and take certain situations for granted. To identify our preconceptions, we strived to build a team of researchers with various backgrounds: two team members worked outside the context under study (a professor in medical education with a background as a veterinarian, and a senior researcher with a clinical background and having the experience of working in non-clinical roles for the past 30 years).

## Results

During our analysis, we identified six strategies the PDs used. We deliberately chose to use the word strategies, because this word most closely matches the wording of the participants in the interviews. PDs’ strategies are presented as themes and compared with the corresponding OS tactics, using the same order as they are mentioned in the paper of Van Maanen and Schein [7] (see Table 3). Then we will provide more detailed descriptions of the strategies, which is a more abstract summation of the issues at hand. The descriptions are supported by illustrative quotes. Finally, we will present two overarching insights we uncovered.

**Table 3:** Comparison between strategies the program director used and the socialization tactics OS

Strategy	Description	Name tactic in OS
Approaching newcomer residents as a group or as individuals	This strategy sets out how PDs focus on both group and individual level. Some PDs actively support the socialization process at group level by organizing group activities. Other PDs observe the socialization process of peer groups of residents without any active involvement. PDs mainly support the socialization process of individuals by focusing on residents with poor performance.	Collective and individual socialization tactic
Facilitating newcomer residents in learning their new role	This strategy describes PDs’ support to residents in learning their new role, which varies from facilitating newcomers with an extensive introduction program to implicit learning of their new role at the workplace. Once an introduction program is implemented, socialization is often an unintentional effect rather than an explicit learning objective.	Formal and informal socialization tactic
Letting newcomer residents get acquainted with many supervisors	This strategy outlines how PDs let newcomers get acquainted with other health care professionals (doctors, nurses, secretary, et cetera). Some PDs actively facilitate direct contact between newcomer residents and other health care professionals. Other PDs do not introduce newcomers to other health care professionals.	Sequential and random socialization tactic
Responding to the development of newcomer residents during their socialization process	This strategy focuses on how PDs let newcomers adjust to their new role over time. Some PDs change the content of the role after a fixed time frame without taking the individual residents’ development into consideration. Other PDs, however, change the content of the role without pre-defining a time frame, taking the residents’ individual development into consideration.	Fixed and variable socialization tactic
Making use of role modeling	This strategy sets out that PDs often make use of role modeling to facilitate the socialization processes of residents. Other health care workers as well as the PDs themselves can be role models. If PDs perceive to be a role model themselves, they vary in the extent to which they make their role modeling behaviors explicit. Unlike OS theory, no examples of socialization without role modeling emerged from the data.	Serial and disjunctive socialization tactic
Acting upon expectations of newcomer residents’ adjustment to their new role	This strategy describes the PDs’ expectations of newcomer residents’ adjustment. Some PDs adapt their approach to fit newcomer residents’ characteristics, others expect newcomer residents to adjust to the (implicit) norms of the workplace.	Investiture and divestiture socialization tactic

### Approaching newcomer residents as a group or as individuals

This strategy describes the way the PDs approached newcomer residents’ socialization. Some PDs separated newcomer residents from their more experienced peers, and other health care professionals. As such, PDs actively contributed to residents’ socialization process, because it appeared as a team building activity.

*‘In the first year they’ve got radiation training, which is a three-and-a-half week course* [which is in another city, so they have to stay overnight] *(…) so then, they definitely get to know each other very well.’ (P12).*

Other PDs acknowledged the importance of collective, group socialization, i.e. as a group of newcomer residents. However, PDs were not actively guiding this socialization process.

*‘Well, I think it’s a sign that the group atmosphere’s just fine (…) I think it’s something that’s just growing and not something we’ve arranged.’ (P7).*

However, the PDs agreed upon the importance of group processes in newcomer residents’ socialization. Since residents worked under similar conditions and faced similar challenges, they could support each other in both work and private situations.

‘*They also do things together. This contributes to an overall feeling of safety and comfort. The group atmosphere’s really important.’ (P14).*

In contrast to approaching newcomers as a group, we also found examples of approaching newcomers as individuals. This approach was mainly used in situations where residents were performing poorly or even failing. As a PD signaled:

‘*I don't see it as my job to listen to what a nurse and a resident are discussing, but I do consider it my job when a resident’s wandering around and not in charge of the processes, failing in the emergency department. Then it’s interesting to find out why it happens and why he gets stuck.*’ *(P13).*

In short, PDs approached residents both at group and individual level. The strategy of approaching newcomers at group level was described as both explicitly organized by the PD and happening by coincidence. In OS, the strategy of approaching newcomers as a group or as individuals is called the collective and individual socialization tactic.

### Facilitating newcomer residents in learning their new role

This strategy includes the way the PDs guided newcomer residents through the process of learning their role. Most PDs offered an introduction program to help newcomer residents to get started and familiarize themselves with their new role. However, only one PD explicitly mentioned that socialization was part of this program.

‘*I made a schedule based on which residents can take a look at different laboratories with various equipment [and process demands] (…) so they can spend half a morning at one laboratory and then half a morning at another, to get to know the people, each other’ (P16).*

In the other interviews, however, PDs stated that socialization was unintentional, even if an introduction program was provided.

Next to a formal introduction program, PDs strategy to take residents through the process of learning their role could often be characterized as facilitating implicit learning. The PDs described that the socialization process just happened naturally:

*‘Ehm, but in an OR complex, you* [residents] *are guided by colleagues and also by experienced nurses, directly, indirectly, directive or in all kinds of different ways (…) and that involves implicit learning. You can’t explain it, but it’s really important (..) So it also happens in the emergency department, I don’t know to which degree, but it happens for sure.’ (P13).*

In summary, PDs’ strategy to facilitate residents in learning their new role varied from a structured introduction program to implicit learning. When an introduction program was offered to newcomer residents, socialization often occurred as an unintentional effect and was rarely mentioned explicitly. In OS, this strategy is called the formal and informal socialization tactic.

### Letting newcomer residents get acquainted with many supervisors

This strategy refers to how PDs let newcomer residents get acquainted with many supervisors. Some PDs organized newcomer residents’ work in such a way that at the beginning they only collaborated with a single or a few supervisors. The PDs did this by delineating their tasks and responsibilities. They increased the complexity of the residents’ tasks and responsibilities over time and, consequently, the number of supervisors increased. In other words, the PDs explicitly structured the process in which residents became acquainted with many supervisors, which is part of the socialization process. This is illustrated by the next quote:

*‘We gradually increase the complexity, for instance, (…) by* [increasing] *the number of supervisors the resident collaborates with on a daily basis. In the beginning they* [the residents] *start with one (job-related task), then they have 2 supervisors, whilst there are 18 in total. (..) Later on, the number of residents’ tasks increases. And, as a consequence, residents have to work with many other supervisors’ (P16).*

Other PDs did not organize the process of getting acquainted with many supervisors. Therefore, newcomer residents had to get used to many new supervisors in a short period of time. The PDs recognized that this was challenging for newcomer residents.

*‘Well, one aspect is: many supervisors, many opinions. (...) With a lot of supervisors, it’s sometimes hard* [for newcomer residents] *to get a little grip* [on the situation]*: one supervisor absolutely does not allow method A, while another insists on using it, so there’s sometimes a difference* [in opinion]*, which can be a bit frustrating. You’re looking for a single recipe* [a standard way of doing things], *and then it takes a while to discover that it can be done in many different ways’ (P1).*

This quote illustrates that socialization occurs in the workplace, however it seems to happen unintentionally. In summary, the strategy of getting acquainted with many supervisors ranged from explicitly organizing the residents’ tasks and responsibilities, through gradually increasing the complexity of their tasks and responsibilities and the number of supervisors over time, to making no arrangement at all. In all situations socialization occurred, intended and unintended. In OS, this strategy is called the sequential and random tactic.

### Responding to the development of newcomer residents during their socialization process

This strategy shows how PDs handle differences in newcomer residents’ development over time. Some PDs changed newcomer residents’ tasks and responsibilities after a fixed time frame and, therefore, did not consider differences in development between residents. As one PD illustrated:

*‘Erm, we’ve a six-week rule, six weeks of introduction, and during this period they work for a couple of weeks in the Cardiac Care Unit* [in which patients with presumed cardiac pathology are diagnosed]*. And in this hospital, residents also do cardiology night shifts at the nursing ward for a couple of weeks because, of course, you have to know a little about how things work there. And they’re in the emergency department for a few weeks.’ (P7).*

Other PDs did not change newcomer residents’ tasks and responsibilities after a fixed time frame but customized supervision based on level of development. In other words, the PD had a flexible time frame for changing the tasks and responsibilities of individual residents. As one PD mentioned:

*‘And we also know* [the resident’s level of performance]. *When we do shifts, you also want to check out the resident you’ll be working with, then you know exactly what the resident can or cannot do. So, it’s not so much that you have to ‘pass that test’, but rather that we adjust the level of supervision to the level of performance of the resident’ (P12)*.

In short, how PDs responded to newcomer residents’ development over time varied from treating every resident the same within a set period of time and changing their tasks and responsibilities accordingly (the transition process has a fixed time frame), to adjusting tasks and responsibilities to fit individual newcomer residents’ development (based on a flexible time frame). In OS, this tactic is called the fixed and variable socialization tactic.

### Making use of role modeling

This strategy refers to how PDs use role modeling to support the socialization process of residents. All PDs mentioned that experienced colleagues, such as senior residents and other health care workers like supervisors, nurses and midwives, served as role models. As a PD stated about senior residents being role models:

*‘Well, you often see, of course, that senior residents already know this* [how things work] *a little bit and they, of course, are a role model for their younger buddies. (‘…’) And in general perhaps a bit more accessible, approachable as a buddy (…).’ (P6).*

Some PDs were aware that residents saw them as a role model. One PD said:

*‘But I, I still value the master-apprentice relationship. I also occasionally do the handover myself. So they have to see how I do it, and they can just mirror me* [my actions or behaviours]*.’ (P5).*

Others assumed they were role models themselves, but did not make it explicit:

‘*There are also residents of whom I think, well, you know, “if I’d do it* [myself]*, it might just go faster” and then, yes, in the weekends, you just want things* [the work] *to be done quickly. An additional advantage is that a resident can also learn something from it.’ (P7).*

In OS, using role modeling in socialization is called the serial tactic. The counterpart (disjunctive tactic) is described as lacking a role model. In the interviews, however, the PDs did not refer to situations where a role model was absent. If PDs acted as role models themselves, they differed in the degree to which they made it explicit for residents.

### Acting upon expectations of newcomer residents’ adjustment to their new role

This strategy shows how PDs varied in their expectations regarding newcomer residents’ adjustment. Some PDs accepted newcomer residents as who they were and gave them positive social support, which eased the transition. As a PD stated:

‘*We have an eye for the vulnerability of young doctors. When it’s really busy, we also make sure that they get compliments. We also try to stimulate them in a positive way, so they don’t have the impression of being* [used as] *a workhorse.’ (P14).*

These PDs adjusted their strategy to individual newcomer resident’ needs.

Other PDs expected residents to adjust to the (implicit) workplace norms and behaviours. A PD stated:

‘*That’s an important thing in [resident] training, (…) is of course that you* [the resident] *are not responsible, right? So you have to learn* [the hierarchy and] *your place as a resident. And* [as a supervisor] *you have to do things like if the resident thinks in one way and I want to do it in another way, then whatever it takes to do it my way must be done. And if that doesn’t happen, the resident gets in.’ (P8).*

In short, PDs differed in their expectations of newcomer residents’ adjustment. Some PDs tailored their strategy to newcomer residents’ needs, whereas others expected newcomer residents to adjust to the (implicit) norms of the workplace. In OS, this strategy is called the investiture and divestiture tactic.

### Overarching insights across strategies

Further inspection of the strategies we identified provided two overarching insights into the way PDs support newcomer residents in their socialization process. The first one refers to the extent to which the socialization process is deliberately planned beforehand (see Table 4). PDs can consider socialization as an explicit learning objective and arrange tasks and responsibilities accordingly. This makes socialization an intended outcome of the program. Alternatively, socialization can happen implicitly as a result of social interactions in the workplace, which is unintentional and may yield various outcomes and side-effects.

**Table 4:** Overarching synthesis across all six socialization strategies: explicit (intentional) learning objectives & implicit (unintentional) effects

Strategy	Socialization as an explicit (intentional) learning objective	Socialization as an implicit (unintentional) effect of social interaction at the workplace
Facilitating newcomers in learning their new role	Making socialization *explicit* in the introduction program	Socialization is an *unintentional* effect of the introduction program
Letting newcomers get acquainted with many supervisors	PDs *explicitly* let newcomers get acquainted with many supervisors	Getting acquainted to other health care professionals is not arranged and therefore socialization occurs *unintentionally*
Making use of role modeling	Making *explicit* to residents that the PD is a role model	Assuming that PDs are a role model for residents without making it explicit and therefore socialization is an *unintentional* effect

The second overarching insight is that the extent to which PDs accommodate newcomer residents’ socialization can vary substantially (see Table 5). PDs tailor their strategies to individual resident’s needs by adjusting the residents’ tasks and responsibilities, thereby creating a certain level of bespoke socialization, particularly around poor performance. In contrast to such individual level strategies, PDs can also employ workplace-centered strategies and expect newcomer residents to adjust to the workplace without much customization, which seems to stem from the implicit expectation that socialization comes naturally.

**Table 5:** Overarching synthesis across all six socialization strategies: individual-centered & workplace-centered approaches to socialization

Strategy	PDs adapt their strategy to individual residents’ needs	PDs expect residents to adjust to the norms of the workplace
Approaching newcomers as a group or as individuals	PDs use an individual strategy for residents with poor performance	PDs expect residents to adjust to their peer group
Responding to the development of newcomers during their socialization process	PDs tailor their strategy to individual residents and have a variable time frame for changing newcomer residents’ tasks and responsibilities	PDs treat every resident the same and change newcomer residents’ tasks and responsibilities after a fixed time frame
Acting upon expectations of newcomer residents’ adjustment to their new role	PDs accept residents’ personal characteristics and adapt their strategy to individual residents’ needs	PDs expect residents to adapt to the (implicit) norms of the workplace

## Discussion

This study aimed to deepen our understanding of what strategies PDs use to facilitate newcomer residents’ transition and guide them through their socialization process. We identified six different strategies, which seemed to correspond with the organizational tactics described by van Maanen and Schein [7]. The overarching insights we uncovered by comparison across strategies showed that PDs strategies varied from mentioning socialization as an explicit (intentional) learning objective to considering socialization as an implicit (unintentional) effect of social interactions at the workplace. Furthermore, PDs differed in using an individual-centered or a more workplace-centered approach to socialization.

Our finding that the PDs’ socialization strategies were often unintentional, resonates with the literature [9, 38]. Hafferty and Castellani stated that socialization often ‘resides at an unconscious or unexamined level to the immediate social actors’ [9]. Therefore it is not surprising that most PDs in our study did not mention socialization as an explicit and intentional learning objective. The observation that socialization occurred despite the absence of socialization as a learning outcome can be explained by the fact that socialization is regarded as one of the driving forces behind what sociologists call ‘social reproduction’ [39, 40]. In other words, socialization is a necessity to sustain the profession, the specialty, the department and the hospital. The observation that socialization occurs unintentionally may be further explained by the fact that existing social structures in the researched context [4, 41–45], such as connection with peers by sharing an office or collaboration with nurses on ward rounds, already propel the socialization of newcomers.

Consequently, it seems that, from a sociological perspective, the use of unintentional strategies can be explained. However, unintentionally using a socialization strategy contrasts with the principles of adult learning. It is essential for adult learners, including residents, to have clear learning goals and objectives [46]. In addition, we did find examples of PDs who already used an intentional and explicit strategy to facilitate the socialization of newcomer residents in transition, but when should PDs and faculty apply intentional strategies? The PDs in our study felt that some residents might struggle with establishing effective working relations with other health care professionals such as supervisors, nurses and peers. In such situations, we recommend to create an intentional strategy for helping residents build relationships to optimize their socialization process.

Our data showed that the PDs’ strategies to foster socialization in residents were closely linked to residents’ daily supervision at work, i.e. clinical supervision in delivering patient care. Therefore, the theoretical perspective of OS offered us a useful conceptual tool to inform our subsequent analysis and a different lens to deepen our understanding and help us make sense of this complex social reality [27]. To smoothen newcomer residents’ transition, it is important to reduce the stress they experience when they have to adapt to their new role within a new context facing challenges like establishing social interaction, mastering new tasks and responsibilities and meeting expectations [5, 6]. The concept of OS differs from the way socialization is often conceptualized in Health Professions Education (HPE) literature. Hafferty [47] distinguished socialization from training by arguing that ‘while any occupational training involves learning new knowledge and skills, it is ‘the melding of knowledge and skills with an altered sense of self that differentiates “training” from “socialization”’. In other words, Hafferty clearly separated socialization from clinical tasks and responsibilities, whereas OS did not distinguish between these processes. Other scholars such as Biesta and van Braak [8] conceptualized socialization as one of the purposes of (medical) education. They differentiated socialization (becoming a member of the professional group), qualification (providing students with knowledge, skills and understanding) and subjectification (becoming a thoughtful, independent, responsible professional), but acknowledged that these aspects overlap. Consequently, the perspective of Biesta and van Braak shares with that of OS that socialization and clinical tasks and responsibilities –what Biesta and van Braak called qualification: the acquisition of knowledge, skills and understanding– overlap and are often intertwined. The difference between these two perspectives lies in their focus. While Biesta and van Braak focused on different educational purposes, OS was developed to focus on socialization in the workplace. In summary, a unifying theory of socialization is lacking; definitions of socialization vary over time and across academic disciplines [14, 47]. In the context of medical education, our deductive analysis through the lens of OS adds to this conversation as it reinforces the notion that socialization and clinical tasks and responsibilities are closely related and often overlap, particularly in newcomer residents.

Although the formal curricula of the researched PGME contexts are built around Competency Based Medical Education (CBME) [48], we found examples in our data contrasting these tenets. One of the characteristics of CBME is using a learner-centeredness approach. Our data provided examples of PDs who adopted a learner-centered socialization strategy by adjusting their strategy to the individual development of the residents, focusing on personal characteristics of the resident and/or supporting poor-performing residents. However, our data also provided examples of PDs who adopted a workplace-centered socialization strategy. These PDs assumed that residents would adapt to their peer group and the implicit norms of the workplace. But, how can we explain these contrasts?

That some PDs preferred a workplace-centered approach to socialization can be explained as follows. Residents do not deliver patient care on their own, or in isolation. Delivering patient care is team work [49]. Therefore, it might be difficult for PDs to discern residents’ individual contributions from the team effort [49]. Moreover, it is not only important that every team member knows how to apply professional standards, it is also important that they are able to function in a team. Socialization is necessary for team work to be effective. Therefore, ‘workplace-centered strategies’–such as expecting residents to adjust to their peer group and to adapt to the (implicit) norms of the workplace (see Table 5)– might even be more important for individual residents, because they need ‘to be able to work in a complex environment in which powerful, often informal, unmentioned, and largely hidden social forces take part’ [46].

### Strengths and limitations

Our decision to apply the lens of the OS tactics to perform a theory-informing inductive data analysis after the data collection and thematic analysis was completed [26], may have prevented us from identifying any examples of the disjunctive tactic. Although our decision to do so can be seen as a limitation since we did not specifically ask for participants’ perceptions of each tactic, all other organizational tactics turned out to be present in the data, suggesting that the OS framework is applicable to our situation. This seems to be supported by van Maanen and Schein [7], who stated that organizational tactics ‘theoretically, at least, can be used in virtually any setting’. If we had chosen a fully theory-informed inductive study design [26] in which the theory informs *every step* of the research process, it would have yielded potentially different outcomes. However, instead of developing or refining a theory, we aimed to broaden our understanding of a situation in clinical practice, in which theory helps to identify ‘processes that occur beneath the surface and so to develop knowledge of underlying (generating) principles’ [27].

Our study focused specifically on organizational tactics, which were also described in the model of Bauer and Erdogan [15, 16] and adapted to fit the transition of undergraduate medical students into clinical clerkship and graduate nurse transition [17, 18]. A strength of our study may be that we conducted a deep exploration of organizational tactics PDs used to facilitate the socialization process of newcomer residents. Based on our results, we propose to slightly modify Bauer and Erdogan’s model by adding to the organizational tactics that the process of socialization can be approached in different ways: as an explicit and intentional learning objective, or as an implicit and unintentional learning objective. Moreover, we could add the nuance that organizational tactics can be individual-centered as well as workplace-centered.

PDs were purposively sampled from different hospitals and different specialties. On the one hand, this can be seen as a strength because we explored the socialization process in different contexts, which may contribute to the transferability [23, 27, 28] of our findings. On the other hand, our sample size is limited and therefore the transferability [23] to other settings is limited. We believe, however, that the results of our study lay a foundation for future research in other settings, such as non-hospital settings and settings in different regions and countries.

We consider the diversity in our research team a strength of our study. Three team members (two residents and one PD) worked within and two outside the researched context. Their different perspectives, gained from lived experiences, helped us make sense of the results [37]. We tried to optimize the quality of our design, analyses and interpretations by adopting a continuous iterative approach, in which we critically reflected on the research process as it developed. The numerous discussions, reflections and conversations may have resulted in a richer overall outcome.

### Future research

We explored how PDs navigated newcomer residents through their socialization process. A next step would be to investigate how residents experience these organizational strategies. From OS literature we know that new situations may cause uncertainty [15] and that newcomer residents may be motivated to reduce these negative effects by learning the ‘functional and social requirements of their newly assumed role as quickly as possible’ [7]. Future research should investigate the effectiveness of the different strategies, specifically the extent to which each strategy affects newcomer residents’ socialization.

Besides, a smooth transition of newcomers into an organization contributes to the continuation of the organization’s mission, values, and performance [7, 14]. Therefore, additional insight into PDs’ socialization strategies would be useful to ease newcomer residents’ transition. Future research involving a large group of PDs and faculty is needed to further unravel the relation between OS tactics, newcomer adjustment, and outcomes [15], and to deepen our understanding of effective organizational support to ease the transition of newcomer residents.

### Practical implications

The results of our study uncovered a broad range of strategies PGME institutions PDs and faculty can use to facilitate the socialization process of newcomer residents. On an institutional level, these strategies can be used to improve PGME standards and inform faculty development courses [50]. On a program level, PDs and faculty can gain valuable insights to facilitate the socialization process of newcomer residents and the possibility of switching between strategies, depending on the situation. They could, for instance, make socialization an intended learning outcome of PGME and/or the introduction program, because clear learning objectives are essential for adult learners like residents [46]. They could also adapt the introduction program by providing ample opportunity for newcomer residents to communicate with their supervisors and build relationships, especially in the beginning of residency training. Residents who struggle with establishing effective working relations with other health care professionals may need additional help. On a supervisor level, the results of our study may create awareness among supervisors of what they could do to ease the socialization process of newcomers. Although research shows that socialization is often an unintentional effect of implicit and informal learning, we argue that PDs should not rely on the implicit expectation that socialization between newcomer residents and supervisors comes naturally in daily practice [50, 51]. As context shapes residents’ learning, we recommend to foster dialogue between newcomer residents, their supervisors, PDs and/or faculty, to discuss the norms and culture of the training program and the PGME institution [51].

## Conclusion

This study empirically illustrates that socialization will occur regardless of which strategy is used. We identified six strategies PDs used in medical practice. Further inspection of these strategies showed that PDs’ strategies may vary from considering socialization as an explicit learning objective to perceiving socialization as an unintentional effect of social interaction in the workplace. Another overarching insight we uncovered was that PDs’ strategies may vary from individual-centered to workplace-centered strategies. Although the workplace-centered strategy contrasts with the learner-centered approach of CBME, it seems essential for the socialization process. The findings of our study may increase the understanding among PGME institutions, PDs and faculty of what can and should be done to positively affect the socialization process of newcomer residents and help them ‘learn the ropes’. Further research is needed to gain a deeper understanding of residents’ perceptions of PD’s efforts to facilitate their socialization process during transitions.

## Supplementary Information

**Additional file 1.** Topic list.

## Data Availability

The datasets used and/or analyzed during the current study are available from the corresponding author on reasonable request.

## Abbreviations

GG: Gerbrich Galema
RD: Robbert Duvivier
JP: Jan Pols
DJ: Debbie Jaarsma
GW: Götz Wietasch
MB: Myrte Boer
CBME: Competency based medical education
HPE: Health Professions Education
OS: Organizational Socialization
PDs: Program directors
PIF: Professional identity formation
PGME: Postgraduate medical education
P1-17: Participant 1-17
