# Supplementary material for: Learning the ropes: strategies program directors use to facilitate organizational socialization of newcomer residents, a qualitative study
Source: BMC Med Educ. 2022 Apr 5;22:247. doi: 10.1186/s12909-022-03315-9 (PMC8981951; doi:10.1186/s12909-022-03315-9)
Supplement: Supplementary file 1 — Additional file 1. Topic list. [file 12909_2022_3315_MOESM1_ESM.docx]

# Additional file 1; topic list

PDs were provided with a background introduction and the goal of the research

1. What is the background of residents when they start with residency (doctor-not-in-training, PhD-trajectory, last clerkship during medicine)?
2. In which situations do you collaborate with residents?
3. Which differences do you acknowledge between an unexperienced and an experienced resident?
   1. Different tasks
   2. Degree of supervision
   3. Clinical tasks
   4. Non-clinical tasks; organization, responsibility, collaborating, communication, learn how to work
4. What happens with a resident in his/her first job?
5. Which growth do you see in the residents’ first period?
